# Case Report: Type B intramural aortic hematoma: conservative management or early intervention? A case of rapid progression to aortic dissection

**DOI:** 10.3389/fcvm.2026.1875853

**Published:** 2026-08-04

**Authors:** Adriana Sbrigata, Laura Asta, Sebastiano Castrovinci, Antonio Segreto, Calogera Pisano

**Affiliations:** 1 Cardiac Surgery Unit, Department of Precision Medicine in Medical Surgical and Critical Area (Me.Pre.C.C.), University of Palermo, Palermo, Italy; 2 Department of Neuroscience, Imaging and Clinical Sciences, Cardiac Surgery Department, University “G. d’Annunzio” Chieti & Pescara, Chieti, Italy

**Keywords:** anticoaugulation, aortic intramural hematoma, pulmonary embolism, thoracic endovascular aortic repair (TEVAR), type B acute aortic syndrome

## Abstract

**Background:**

Intramural hematoma (IMH) is a variant of acute aortic syndrome (AAS) with a highly variable and potentially life-threatening course. Although uncomplicated type B IMH is generally managed conservatively, early disease progression may occur despite optimal medical therapy. Thoracic endovascular aortic repair (TEVAR) represents a safe and effective treatment option in complicated cases, particularly when timely intervention is achieved.

**Case presentation:**

A 69-year-old woman presented with acute chest pain radiating to the back. CT angiography revealed a type B IMH extending from the aortic arch to the celiac trunk. The patient was initially managed medically under close surveillance. During hospitalization, she experienced recurrent chest pain and was incidentally diagnosed with a subsegmental pulmonary embolism, for which low-molecular-weight heparin was initiated Initial follow-up imaging showed no disease progression. However, on day 7, worsening symptoms prompted repeat CT, which demonstrated significant IMH progression with early signs of type B aortic dissection. Emergency TEVAR was successfully performed. The postoperative course was uneventful except for a transient post-implantation syndrome. Follow-up imaging confirmed good stent graft positioning and significant reduction of the hematoma. This case highlights the unpredictable evolution of type B IMH and emphasizes the importance of close clinical and radiological surveillance. It also illustrates the therapeutic dilemma posed by concomitant pulmonary embolism requiring anticoagulation.

## Case report

**Figure 1:**
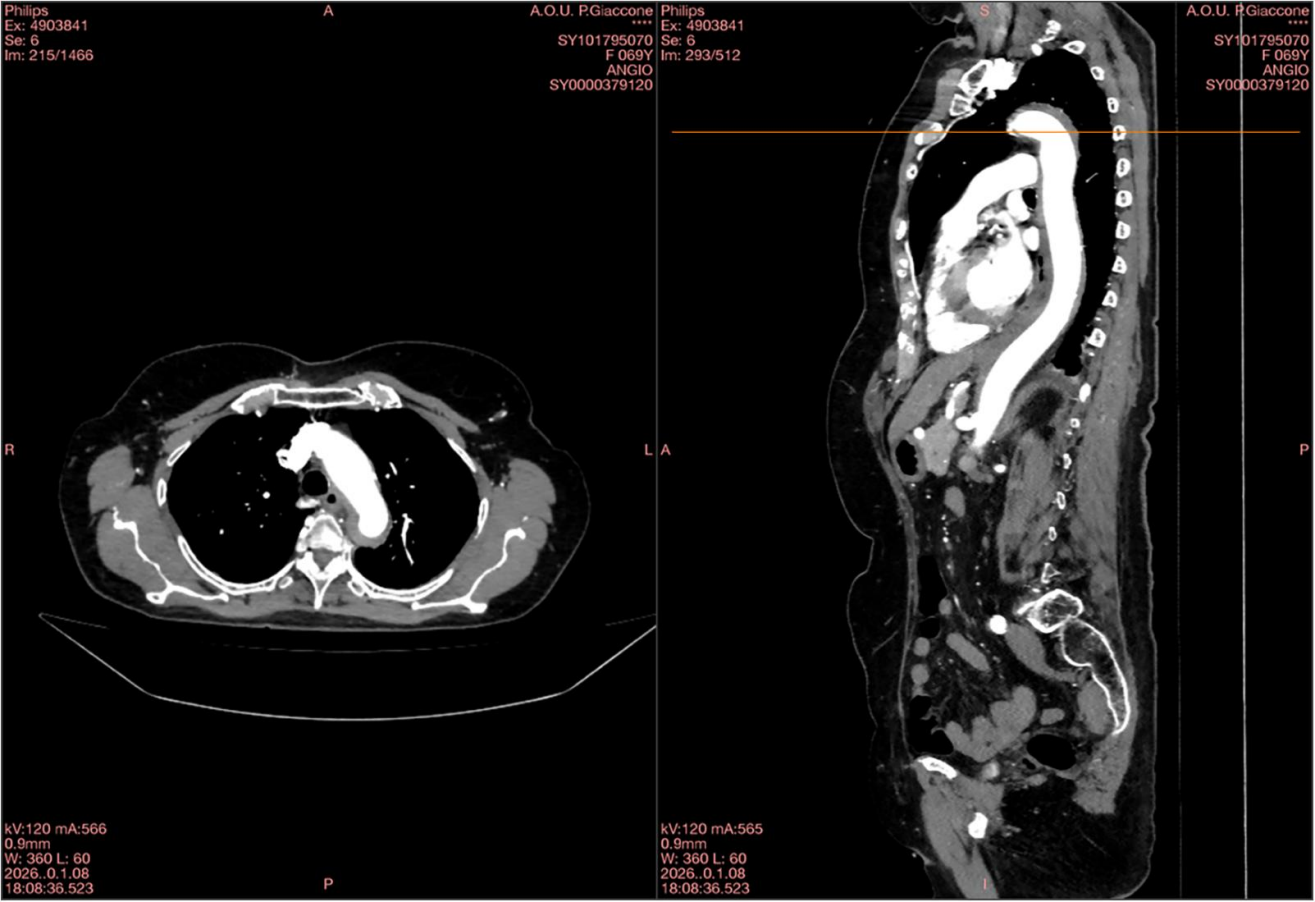
Initial CT angiography showing IMH extending from the mid aortic arch to the origin of the celiac trunk.

A 69-year-old woman presented to the emergency department with stabbing chest pain radiating to the back. Preoperative evaluation revealed a history of hypertension and gastroesophageal reflux disease, treated with an angiotensin receptor blocker, beta-blocker, and proton pump inhibitor. The patient was a non-smoker, had no relevant family history, and was not receiving anticoagulant or antiplatelet therapy. On admission, the patient was hemodynamically stable, with unremarkable physical examination findings. Routine blood tests, renal and liver function, cardiac biomarkers, and transthoracic echocardiography were unremarkable, whereas serial inflammatory and coagulation markers progressively increased of C-reactive protein, fibrinogen, and D-dimer levels: serial D-dimer levels progressively increased from 1,223 to 4,405 and 7,246 ng/mL FEU, accompanied by increasing CRP (9→43→47 mg/L) and fibrinogen (321→545 mg/dL). Acute coronary syndrome was initially considered in the differential diagnosis but was excluded based on negative cardiac biomarkers and a normal electrocardiogram. In the Emergency Department, thoracoabdominal CT was performed, followed by contrast-enhanced CT angiography. Imaging demonstrated an aortic arch intramural hematoma with a maximum thickness of 8 mm extending from the mid-aortic arch to the origin of the celiac trunk (Figure 1). The patient was then referred to our Cardiac Surgery Unit. Table 1 defines the complete timeline of events that characterized the patient's clinical course.

**Table 1:** Clinical timeline of the patient's course.

Time	Clinical event
Day 0	Presentation to the Emergency Department with acute stabbing chest pain radiating to the back.
Day 0	Electrocardiogram and cardiac biomarkers excluded acute coronary syndrome. CT angiography demonstrated a type B intramural hematoma (IMH) extending from the mid aortic arch to the origin of the celiac trunk (maximum thickness 8 mm).
Day 0	Admission to the Cardiac Surgery Unit. Conservative management initiated with blood pressure control, pain management, and close clinical and radiological surveillance.
Days 1–5	Recurrent episodes of chest and interscapular pain. Serial CT angiography showed no significant changes in the IMH.
Day 5	Follow-up CT angiography incidentally detected a segmental pulmonary embolism in the left upper lobe. Therapeutic low-molecular-weight heparin (LMWH) was initiated.
Day 7	Follow-up CT angiography demonstrated complete resolution of the pulmonary embolism and stability of the IMH.
Day 7 (+6 h)	Clinical deterioration with recurrent chest pain. Emergency CT angiography revealed rapid IMH progression (maximum thickness 18 mm) with imaging features suggestive of impending type B aortic dissection.
Day 7	Emergency thoracic endovascular aortic repair (TEVAR) successfully performed using two overlapping thoracic stent grafts.
Postoperative period	Uneventful recovery except for transient post-implantation syndrome, successfully treated with NSAIDs.
Postoperative day 14	Follow-up CT angiography confirmed correct stent graft positioning and marked regression of the IMH. Patient discharged home.

Over the following days, the patient experienced recurrent episodes of chest pain radiating to the interscapular region, occasionally associated with epigastric pain. The episodes resolved spontaneously or after paracetamol administration, while serial CT scans demonstrated no significant changes in the IMH. Five days after admission, repeat CT angiography incidentally revealed a segmental pulmonary embolism in the left upper lobe. The patient remained asymptomatic, without dyspnea, and echocardiography showed no evidence of right ventricular strain. In addition, compression ultrasonography of the lower limbs showed no evidence of deep vein thrombosis. Anticoagulation with low-molecular-weight heparin (LMWH) was therefore initiated. Approximately 48 h after LMWH initiation, surveillance CT angiography demonstrated complete resolution of the pulmonary embolism and stable IMH. No additional etiological work-up was performed during hospitalization. Medical management consisted of strict blood pressure control with continuation of the patient's antihypertensive therapy, proton pump inhibitor therapy, intravenous fluids, and multimodal analgesia with intravenous paracetamol and sublingual sufentanil. Therapeutic low-molecular-weight heparin was initiated immediately after the incidental diagnosis of pulmonary embolism on hospital day 5. Perioperative antibiotic prophylaxis consisted of cefazolin.

**Figure 2:**
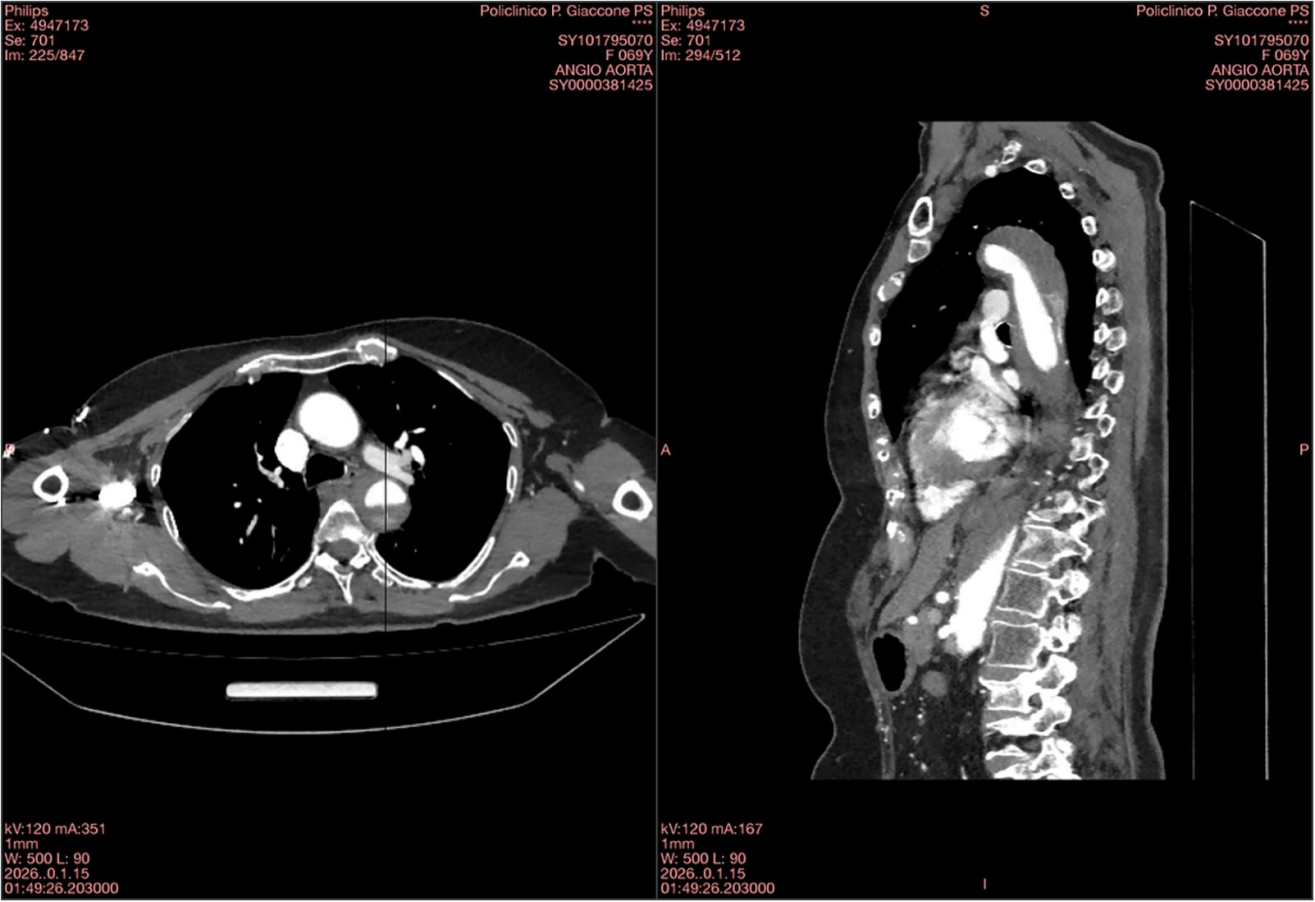
CT angiography on day 7 showing IMH progression with early signs of type B aortic dissection.

Six hours later, repeat CT demonstrated marked IMH progression, with maximal thickness increasing to 18 mm immediately distal to the left subclavian artery and imaging features consistent with early evolution toward type B aortic dissection (Figure 2).

**Figure 3:**
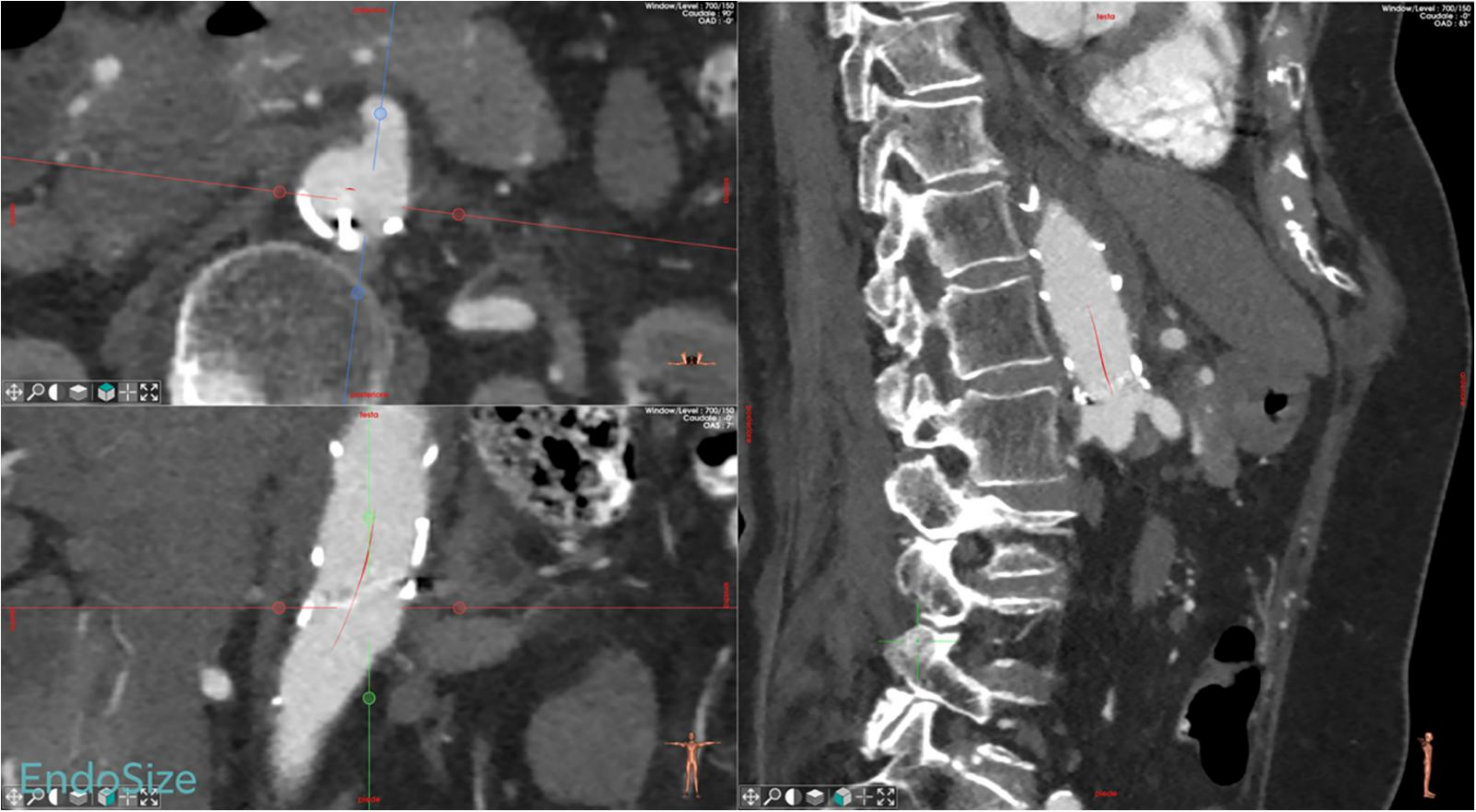
Intraoperative angiography demonstrating preserved perfusion of the left carotid artery and celiac trunk.

An emergency thoracic endovascular aortic repair (TEVAR) was performed under general anesthesia with fluoroscopic guidance. Two Bolton Medical Relay Pro Thoraflex stent grafts (32/28–259 mm and 34/30–154 mm) were successfully deployed with an overlapping configuration. Final angiography confirmed proper device positioning with preserved perfusion of the left carotid artery and the celiac trunk (Figure 3).

The postoperative course was uneventful, with no major complications. The patient developed hyperpyrexia of unknown origin, with negative microbiological cultures and elevated inflammatory markers, likely attributable to post-implantation syndrome (PIS), a systemic inflammatory response to endovascular grafting described in the literature. Nonsteroidal anti-inflammatory drug (NSAID) therapy was initiated with clinical improvement.

The patient was discharged on postoperative day 14.

At discharge, the patient received a proton pump inhibitor, angiotensin receptor blocker, beta-blocker, low-dose aspirin, fondaparinux for 30 days because of the recent pulmonary embolism, NSAIDs for 15 days, and oral iron supplementation.

**Figure 4:**
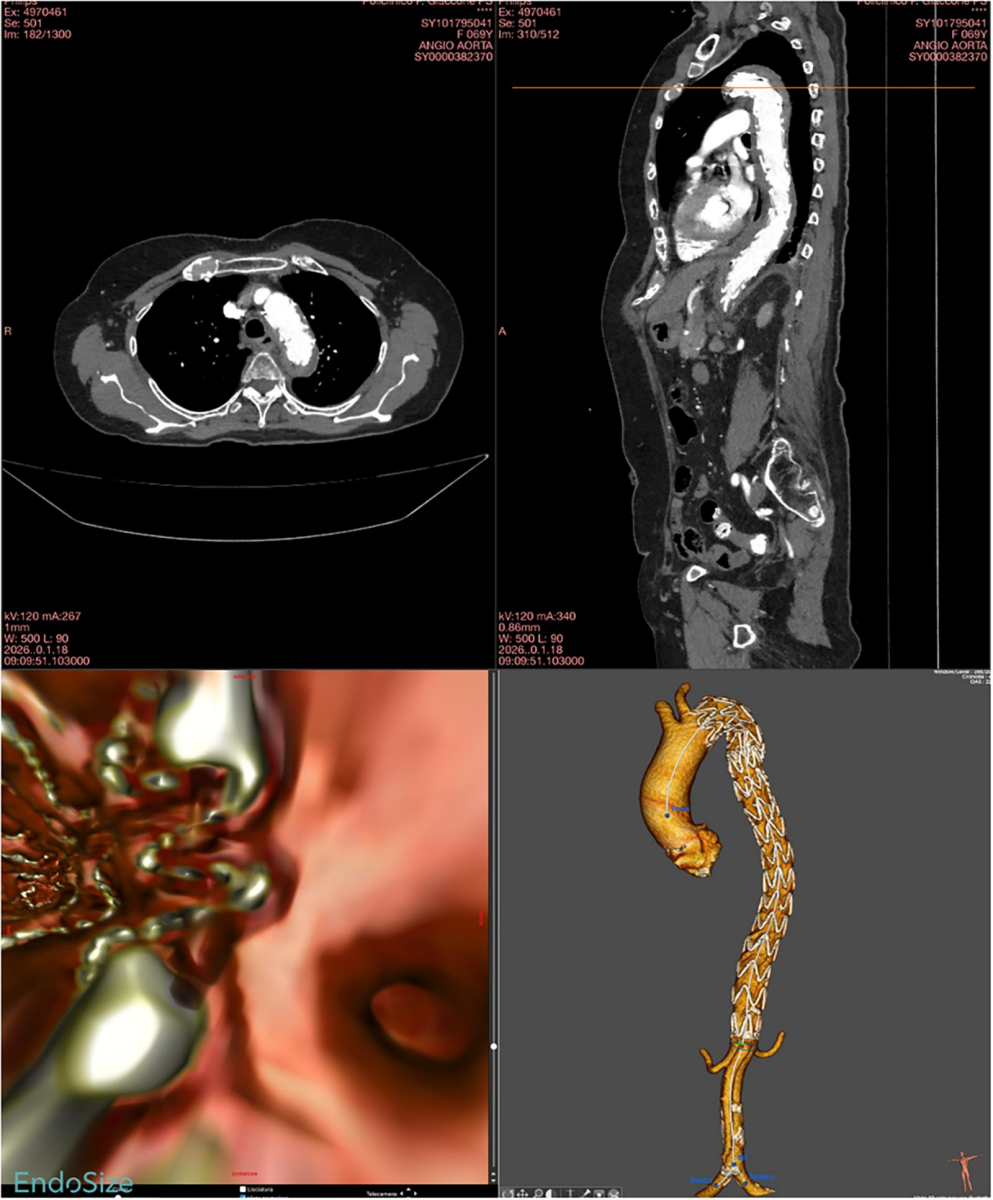
Follow-up CT scan and 3D reconstruction of TEVAR using EndoSize software.

At the two-month follow-up, the patient remained asymptomatic, inflammatory markers had normalized, and CT angiography confirmed good sealing of the stent grafts and a marked reduction in IMH thickness, with a residual subcentimeter diameter (Figure 4). Annual thoracoabdominal CT angiography and cardiac surgical follow-up were recommended.

## Discussion

IMH is part of the spectrum of acute aortic syndromes (AAS) and consists of hemorrhage within the aortic media, with variable thickness and longitudinal extension (1). It is a potentially life-threatening condition with a highly variable clinical course: it may regress, remain stable, or progress to aortic rupture or intimal disruption, leading to overt aortic dissection (2).

Clinical presentation depends on the location and extent of the lesion. Chest pain is the most common symptom. In type A IMH, pain typically radiates to the interscapular region, whereas type B IMH more often presents with severe back pain (3).

IMH remains frequently underdiagnosed and is associated with a reported mortality rate ranging from 10% to 50% with progression to acute dissection in more than 40% of cases. The descending thoracic aorta is the most common site (type B IMH, 60%–70%), followed by the ascending aorta (30%) and the aortic arch (10%).

CT angiography represents the gold standard for diagnosis, risk stratification, and treatment planning in aortic diseases (2, 4). IMH typically appears as a crescent-shaped, hyperattenuating thickening of the aortic wall (>7 mm) without contrast enhancement or an identifiable intimal flap (5).

The natural history of type B IMH is heterogeneous. Although many patients remain stable or experience complete regression with optimal medical therapy, approximately 15%–30% progress to classic aortic dissection, while up to one-third develop aortic-related adverse events during follow-up, including aneurysmal degeneration or the need for intervention. Reported rates of TEVAR vary widely among published series (approximately 20%–30%) because treatment is generally reserved for patients who develop complications such as persistent or recurrent pain, hematoma expansion, ulcer-like projection, impending rupture, or overt dissection (6).

To our knowledge, the coexistence of pulmonary embolism (PE) and acute aortic syndrome is exceptionally uncommon, with the available evidence limited to isolated case reports. Kim et al. described a patient who developed a type B intramural hematoma (IMH) shortly after thrombolysis and anticoagulation for massive PE, suggesting that rupture of the aortic vasa vasorum may have been precipitated by antithrombotic therapy (7). Anticoagulation was discontinued, an inferior vena cava filter was inserted, and the IMH resolved with conservative treatment. More recently, Gouran et al. reported simultaneous acute type B aortic dissection and PE, in which right ventricular enlargement and dysfunction on transthoracic echocardiography prompted further investigation and the diagnosis of concomitant PE (8). Del Monaco et al. subsequently described an iatrogenic thoracic IMH following pacemaker implantation complicated by acute PE, successfully managed conservatively with serial CT surveillance (9).

Our case differs substantially from these reports. Unlike Kim et al., IMH preceded anticoagulation and represented the primary pathology. Unlike Gouran et al., PE was not suspected from the initial presentation but was detected incidentally during surveillance imaging in the absence of respiratory symptoms or right ventricular strain. Furthermore, unlike Del Monaco et al., our patient had a spontaneous type B IMH rather than procedure-related aortic injury. Most importantly, shortly after initiation of therapeutic anticoagulation for PE, the IMH rapidly progressed toward overt type B aortic dissection, ultimately requiring emergency thoracic endovascular aortic repair (TEVAR). Although causality cannot be inferred from a single case, this temporal sequence underscores the complex therapeutic dilemma posed by concomitant PE and IMH.

Taken together, the available literature suggests that concomitant PE and IMH may occur in different clinical settings, but evidence to guide management remains extremely limited. Treatment should therefore be individualized through multidisciplinary discussion, carefully balancing the risk of thromboembolic events against the potential for progression of acute aortic disease (10).

According to the most recent ESC guidelines (2024), initial management of uncomplicated type B IMH includes optimal medical therapy with strict blood pressure and heart rate control, along with adequate pain management and close imaging surveillance.

Initially, anticoagulation was withheld because of concerns regarding the risk of intramural hematoma (IMH) progression. However, the incidental diagnosis of pulmonary embolism necessitated the initiation of therapeutic low-molecular-weight heparin (LMWH). Although a causal relationship cannot be established, the close temporal association between the start of anticoagulation and the subsequent rapid progression of the IMH raises the possibility that LMWH may have contributed to hematoma expansion in a patient with an already unstable aortic lesion. Alternatively, the progression may simply reflect the natural history of a high-risk IMH, as our patient also exhibited recognized predictors of adverse evolution, including persistent chest pain and progressive inflammatory activation. This case highlights the therapeutic dilemma posed by concomitant venous thromboembolism and acute aortic syndrome, where the benefits of anticoagulation must be carefully balanced against the potential risk of aortic disease progression. Further evidence is needed to guide the management of these challenging clinical scenarios.

Although inferior vena cava filter placement could theoretically represent an alternative in patients with contraindications to anticoagulation, it was not considered appropriate in our patient because anticoagulation was not absolutely contraindicated and the pulmonary embolism was hemodynamically insignificant.

The distinction between uncomplicated and complicated IMH is based on the presence of recurrent pain, hematoma expansion, periaortic hemorrhage, or intimal disruption. In uncomplicated cases, serial CT imaging is recommended to monitor disease evolution. If indicated, delayed endovascular treatment may be considered in the subacute phase (14–90 days). In contrast, complicated type B IMH requires prompt intervention with TEVAR (4).

In cases of hemodynamic instability or impending rupture, endovascular repair is the preferred approach. Reported mortality rates are significantly lower for TEVAR (7.2%) compared to open surgical repair (15.9%) in the acute setting. Current consensus supports TEVAR as first-line therapy for descending aortic AAS when anatomically feasible, reserving open surgery for selected patients with unsuitable anatomy or acceptable surgical risk (5).

## Conclusion

This case highlights the unpredictable behavior of type B IMH, which may rapidly progress despite initially stable imaging findings and optimal medical therapy. Careful clinical assessment combined with serial CT surveillance is essential to identify early disease progression and allow timely intervention. When complications develop, TEVAR represents the preferred treatment strategy and may prevent catastrophic aortic events.

## Data Availability

The original contributions presented in the study are included in the article/Supplementary Material, further inquiries can be directed to the corresponding author.
