# Supplementary material for: Case Report: Type B intramural aortic hematoma: conservative management or early intervention? A case of rapid progression to aortic dissection
Source: Front Cardiovasc Med. 2026 Aug 4;13:1875853. doi: 10.3389/fcvm.2026.1875853 (PMC13481195; doi:10.3389/fcvm.2026.1875853)
Supplement: Supplementary file 1 [file Table1.docx]

# CARE Checklist

Manuscript title: Type B intramural aortic hematoma: conservative management or early intervention? A case of rapid progression to aortic dissection

| CARE Item | Description | Location in manuscript |
| --- | --- | --- |
| 1. Title | Diagnosis/intervention and 'case report' in title | Title |
| 2. Keywords | 2–5 keywords | Keywords |
| 3a. Abstract | Background and rationale | Abstract – Background |
| 3b. Abstract | Clinical presentation | Abstract – Case presentation |
| 3c. Abstract | Diagnosis/intervention/outcome | Abstract – Case presentation |
| 3d. Abstract | Main lesson | Abstract – final sentence |
| 4. Introduction | Brief background | Discussion (opening paragraphs) |
| 5a. Patient information | Demographics | Case Report |
| 5b. Patient information | Chief complaint | Case Report |
| 5c. Patient information | Medical/family history, medications | Case Report |
| 6. Clinical findings | Physical examination | Case Report |
| 7. Timeline | Chronological timeline | Table 1 |
| 8a. Diagnostic assessment | Diagnostic methods | Case Report |
| 8b. Diagnostic assessment | Diagnostic challenges | Not applicable |
| 8c. Diagnostic assessment | Diagnostic reasoning/differential diagnosis | Case Report |
| 8d. Diagnostic assessment | Prognostic characteristics | Not applicable |
| 9a. Therapeutic intervention | Type of intervention | Case Report |
| 9b. Therapeutic intervention | Administration/procedure | Case Report |
| 9c. Therapeutic intervention | Changes with rationale | LMWH initiation; Emergency TEVAR |
| 10a. Follow-up and outcomes | Clinical course | Case Report |
| 10b. Follow-up and outcomes | Follow-up tests | Case Report |
| 10c. Follow-up and outcomes | Intervention tolerability | Postoperative course |
| 10d. Follow-up and outcomes | Adverse events | Post-implantation syndrome |
| 11a. Discussion | Strengths/limitations | Discussion |
| 11b. Discussion | Relevant literature | Discussion |
| 11c. Discussion | Scientific rationale | Discussion |
| 11d. Discussion | Take-away lessons | Conclusion |
| 12. Patient perspective | Patient perspective | Not obtained |
| 13. Informed consent | Written informed consent | Consent for publication |
